# Updates on drug discovery in ovarian cancer

**DOI:** 10.1186/2053-6844-1-3

**Published:** 2014-09-30

**Authors:** Steven J Gibson, Krishnansu S Tewari, Bradley J Monk, Dana M Chase

**Affiliations:** The Division of Gynecologic Oncology, University of Arizona Cancer Center, 500 West Thomas Road, Suite 600, Phoenix, AZ 85013 USA; Creighton University School of Medicine, St. Joseph’s Hospital and Medical Center, A Dignity Health Member, 500 West Thomas Road, Suite 600, Phoenix, AZ 85013 USA; The Division of Gynecologic Oncology, Department of Obstetrics & Gynecology, The Chao Family Comprehensive Cancer Center, University of California, Irvine Medical Center, 101 The City Drive South, Building 56, Room 275, Orange, CA 92868 USA

**Keywords:** Ovarian cancer, Chemotherapy, Targeted therapy, Angiogenesis, Recurrent cancer, Clinical trials

## Abstract

Drug discovery in the ovarian cancer arena continues to launch important new clinical trials. Many biologic agents are being studied in phase II and phase III clinical trials for recurrent disease. These agents include compounds that disrupt angiogenesis through a variety of mechanisms. Other oncogenic pathways are also specifically targeted such as PARP, MEK, and topoisomerase inhibitors which are currently being studied in phase III trials. Various cytotoxic agents, as well as therapeutic vaccines, are also under investigation, and continue to demonstrate promising new data. The relevant agents in the treatment of ovarian cancer which have demonstrated positive phase II activity will be discussed.

**Electronic supplementary material:**

The online version of this article (doi:10.1186/2053-6844-1-3) contains supplementary material, which is available to authorized users.

## Review

Remissions after primary therapy in ovarian cancer are usually short-lived. Although intially responsive to a platinum and taxane-based therapy, recurrent disease is difficult to treat. Furthermore, there are few approved agents to treat recurrent ovarian cancer. Although patients that recur after 6 to 12 months of initial treatment may be retreated with a platinum plus taxane, those who relapse earlier or develop significant toxicity, may be given pegalated lipsomal doxorubicin, gemcitabine (in combination with platinum), etoposide, alkeran, topotecan, and/or hexamethylmelamide [1]. Unfortunately the response rate to these agents is generally less than 30%, and demonstrable survival benefits have not been shown. With the introduction of targeted drugs, such as trastuzumab in breast cancer, strategies in drug development have focused on the development of biologic agents that demonstrate selectivity for tumor tissue.

## Introduction

In 2010, we published on recent advances in drug discovery for ovarian cancer [1]. Since then, multiple drugs have either failed to advance into further development, have newly been developed, or have demonstrated activity in phase III trials. For example, with respect to bevacizumab, several positive phase III trials have supported the use of this drug in upfront and recurrent ovarian cancer cases yet FDA approval is pending. Another example includes a 940 patient, phase III AGO-OVAR16 study which proved advantageous in ovarian cancer treatment with pazopanib, increasing median progression-free survival (PFS) by about 5.6 months [2]. In addition, trabectedin was previously discussed and positive phase III activity was reported, improving PFS, and overall response rate in a 672 patient study [3]. Lastly, phase III results from the TRINOVA-1 trial of over 900 patients found that trebananib (AMG 386) increased PFS as well as reduced disease progression and death by 34% when combined with paclitaxel. Unfortunately, several of the drugs previously described have been found to be inactive, or with disappointing clinical outcomes. This review will thus highlight new drugs for ovarian cancer that have recently demonstrated positive phase II activity (Table 1). The ultimate goal with this type of drug development is to achieve prolonged remission and improved quality of life (QOL), for patients with recurrent ovarian cancer.

**Table 1:** **Updates in ovarian cancer drug discovery demonstrating positive phase II activity**

Category		Agent/patent (manufacturer)	Mechanism of action
Targeted agents	VEGF-dependent angiogenesis inhibition	**Cediranib** (AZD2171)/US20070135462 (AstraZeneca)	Oral VEGFR-1,-2,-3 tyrosine kinase inhibitor
		**Nintedanib** (BIBF1120)/US20140018405 (Boehringer Ingelheim)	Oral VEGFR, PDGFR, FGFR tyrosine kinase inhibitor
	PARP inhibitors	**Olaparib** (AZD2281)/US2010098763 (AstraZeneca)	Oral Poly (ADP-ribose) polymerase-1,-2 inhibitor
		**Rucaparib** (CO-338)/US8765751 (Clovis Oncology)	Oral Poly (ADP-ribose) polymerase-1,-2 inhibitor
		**Niraparib** (MK-4827)/US20120035244 (Merck)	Oral Poly (ADP-ribose) polymerase-1,-2 inhibitor
	MEK inhibitors	**Selumetinib** (AZD6244)/US8193229 (Array BioPharma/AstraZeneca)	Oral inhibitor of MEK-1,-2
		**Binimetinib** (MEK162)/US20130273061 (Array BioPharma/Novartis)	Oral inhibitor of MEK-1,-2
Cytotoxic agents		**Etirinotecan pegol** (NKTR-102)/US20090074704 (Nektar)	Inhibits Topoisomerase I (IV)
		**Paclitaxel poliglumex** (CT2103)/US20070167349 (CTI BioPharma)	Mitotic inhibitor (IV)
		**Lurbinectedin** (PM1183)/US20130266666 (PharmaMar)	Marine-derived DNA minor groove binder (IV)
Therapeutic vaccines		**Catumaxomab**/US20120095192 (Trion Pharma)	Oral tri-functional antibody binds EpCAM, CD3, and Fc receptor

## Targeted agents

### Angiogenesis inhibitors

#### VEGF-dependent

**Figure 1:**
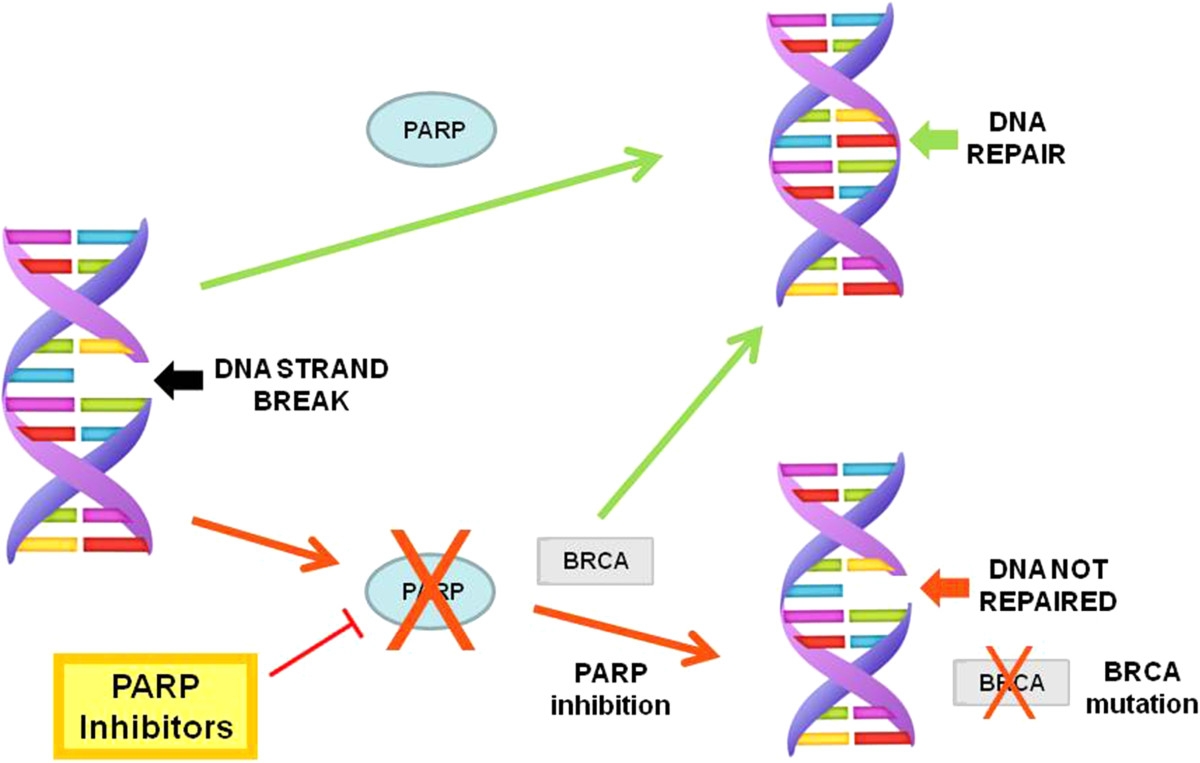
**Poly(ADP-Ribose) polymerase (PARP) inhibitors.**

Vascular endothelial growth factor (VEGF) is a signaling molecule involved in triggering the growth of blood vessels within cancers. The VEGF mechanism of action encompasses binding to tyrosine kinase transmembrane receptors (VEGFR), found on tumor endothelial cells, initiating angiogenesis (Figure 1) [4]. VEGFR-2 regulates cellular VEGF interactions, making it a crucial component in the angiogenic process. Modulating VEGF has become a highlighted area of study with potential in therapeutic interventions.

#### VEGFR-1,-2,-3 inhibitor: cediranib (AZD2171)

While multiple phase III trials of cediranib (Recentin™) may have been disappointing for colon cancer drug discovery, the drug has gained noticeable attention in the treatment of ovarian cancer [5, 6]. Cediranib is a potent anti-angiogenesis agent that acts by blocking the VEGF signaling cascade via the inhibition of all three VEGFR tyrosine kinases, thus preventing the formation of tumor vasculature [7].

Cediranib was evaluated in combination with the PARP inhibitor, olaparib, in the first ovarian cancer trial to utilize two orally-administered investigational drugs [8]. The multi-center, phase II study comparing olaparib (400 mg twice daily) to cediranib (30 mg daily) plus olaparib (200 mg twice daily) found that the combination had nearly doubled PFS (9 vs 17.7 months). Of the 90 platinum-sensitive or BRCA-mutation patients in the study, significantly more had an objective response rate (ORR) in the combination group (56% vs 84%). With these improvements in PFS and ORR came an increase in grade 3/4 toxicities, with about ten times as many observed with the combination (7% vs 70%) [9]. The ICON6 phase III European trial found similar improvements in PFS and overall survival (OS) when cediranib was combined with platinum-based chemotherapy [10]. With these encouraging results, the planning of a phase III cediranib and olaparib combination trial is underway [8].

#### VEGF receptor, platelet-derived and fibroblast growth factor receptor: Nintedanib (BIBF 1120)

The additional targeting of proangiogenic receptors continues to be of interest, and has been proposed to improve the efficacy of VEGF blockade. Nintedanib is a triple angiokinase inhibitor that simultaneously blocks the VEGF, platelet-derived, and fibroblast growth factor receptors [11]. When studied in animal tumor models, nintedanib effectively reduced tumor blood vessel density and integrity [12].

A randomized, placebo-controlled phase II trial evaluated nintedanib maintenance therapy (250 mg for 36 weeks), after chemotherapy in patients with relapsed ovarian cancer. Eighty-three women were enrolled and following the treatment cycle, the PFS was 16.3% for nintedanib patients and 5% for placebo patients. Nintedanib patients experienced a much higher rate of grade 3 or 4 hepatotoxicity (51.2%), compared to that of the placebo group (7.5%) [13]. The potential effect of nintedanib nearly tripling PFS, when compared to the placebo, has warranted a 1,300 patient, phase III study of this drug in the LUME-Ovar 1 trial [14]. Phase III trials of nintedanib are also currently underway for non-small cell lung cancer and being planned for hepatocellular, renal, and colorectal cancers [12].

### Poly (ADP-ribose) polymerase inhibitor

#### Olaparib (AZD2281)

Poly(ADP-ribose) polymerases (PARPs) are proteins involved in the repair of DNA [15]. PARPs assist in the repair of DNA single-strand breaks by repairing base excisions. BRCA1 and BRCA2 proteins involved in DNA recombination play crucial roles in repairing double-strand breaks, and can do so in the setting of PARP inhibition. However, PARP inhibition in BRCA-deficient cells results in the incapability to repair DNA damage induced by chemotherapy. In a BRCA-deficient environment, cell death can manifest when DNA breakage is not repaired, and the cell is exposed to such agents as PARP inhibitors that hinder single-strand break repair (Figure 1) [16].

BRCA mutations represent a minority of breast and ovarian cancers. These homozygous mutations, that are unique to the tumor cells, result in the inability to repair DNA which then can be exclusively targeted by a PARP inhibitor, preserving the patient’s non-tumor cells. Preclinical studies discussed by Fong et al. [17] demonstrate that BRCA-deficient cells were 1000-fold more sensitive to PARP inhibitors. Ledermann et al. supported these findings in subgroup analysis of a 265 patient study evaluating olaparib (400 mg) maintenance therapy in platinum-sensitive, relapsed ovarian cancer. The original phase II study found PFS to be significantly longer with olaparib maintanence than placebo (8.4 vs. 4.8 months, respectively) [18]. A preplanned, subgroup analysis showed patients with a BRCA mutation (BRCAm) had the greatest clinical benefit, specifically germline BRCAm which had a 7.1 month increase in PFS and significant improvement in QOL. They found an average 6.9 month increase in PFS, an 8.5 month improvement in time to second disease progression, and a three month increase in OS in BRCAm patients when compared with placebo [19].

These results have initiated two phase III (SOLO) trials [20]. The U.S.-based SOLO1 trial, will examine olaparib (300 mg twice daily) as maintenance therapy in 2,500 BRCAm ovarian cancer patients following first-line platinum chemotherapy [21]. The European-based SOLO2 trail will examine the same dosage of olaparib maintenance therapy but instead in 440 BRCAm patients with platinum-sensitive, relapsed, or recurrent ovarian cancer [22].

A separate, randomized, phase II study also found significant improvement in PFS with the addition of olaparib to paclitaxel and carboplatin followed by subsequent maintenance therapy (12.2 vs. 9.6 months placebo). The ORR remained fairly similar (64% vs. 58%), and the most common adverse events reported in the combination phase were alopecia, nausea, and fatigue [23].

#### Rucaparib (CO-338)

Rucaparib is an inhibitor of poly(ADP-ribose) polymerases 1 and 2, and works via the same mechanism as olaparib described above and illustrated in Figure 1. Data suggests that PARP inhibitors are especially effective in patients with mutations in DNA repair mechanisms, such as those carrying BRCA mutations [24]. The ARIEL2 trial is investigating specific biomarkers like these to evaluate which, if any, subgroups of the 180 ovarian cancer patients may be sensitive to rucaparib treatment [25]. This data will be incorporated into ARIEL3, a current, randomized, double-blind study phase III trial comparing rucaparib treatment to placebo in 540 ovarian cancer patients [26].

#### Niraparib (MK-4827)

Niraparib, like the other PARP inhibitors above in Figure 1, blocks poly(ADP-ribose) polymerases 1 and 2 from repairing single-strand breaks in damaged DNA. While most PARP inhibitors exhibit similar catalytic inhibitory properties, it is important to note the differences in potency among drugs in this class [27]. Many propose this discrepancy is due to each drug’s individual ability to trap the PARPs at the damaged DNA site. Interestingly, the trapped PARP-DNA complex that forms is more cytotoxic than the single-strand DNA break itself, associating the potency of PARP inhibitors to their ability to form these complexes [27, 28]. Murai, et al. demonstrated that niraparib was the most potent of the PARP inhibitors due to its superior ability in forming the trapped PARP-DNA complex [28].

A large, 100 patient, phase I trial evaluated niraparib in BRCA 1/2 mutation-carrying (BRCA-MC) patients with high-grade serous ovarian cancer (HGSOC) (n = 49), among others cancers. Of these ovarian cancer patients, 45% had a partial response from niraparib (60 mg daily). The platinum-sensitive HGSOC subgroup had a response rate almost double that of the platinum-resistant group (60% vs. 33%, respectively), with a median time of response of 429 compared to 340 days. The most common adverse events were grade 1/2 anemia (48%), fatigue (42%), nausea (42%), and thrombocytopenia (35%). The maximum tolerated dose was determined to be 300 mg daily [29]. Niraparib is currently being studied as maintenance therapy in the randomized, phase III NOVA trial evaluating daily niraparib (300 mg) in 360 patients with high grade serous, platinum sensitive, relapsed ovarian cancer [30].

### MAPK kinase (MEK-1 and -2) inhibitors

#### Selumetinib (AZD6244)

Selumetinib is a mitogen-activated protein kinase inhibitor that shows preclinical benefit in targeting the MEK oncogenic pathway. The small molecular agent is a protein regulator in activated oncogenic pathways expressed in ovarian cancer patients. Results from a phase II study indicate positive activity in the treatment of ovarian cancer. Fifty-two women received two doses of selumetinib (100 mg daily) in the clinical trial, and grade 4 adverse events were only observed in 3 patients (6%). Thirty-four (63%) of the women in the study had a PFS of more than 6 months, with a median OS of 11 months [31]. Larger phase III studies are being planned to further investigate the use of selumetinib as a viable treatment option in ovarian cancer.

#### Binimetinib (MEK162)

Binimetinib is an oral inhibitor of MEK-1 and MEK-2, both of which play an important role in cancer cell proliferation and survival via the RAS/RAF/MEK/ERK signal cascade. Inhibiting this pathway is believed to interrupt growth-factor mediated cell signaling as well as inhibit the production of inflammatory cytokines [32]. Binimetinib is currently the subject of nearly twenty clinical trials, including three phase III trials in ovarian cancer and melanoma [33]. The MILO study is an international, randomized phase III study seeking to compare binimetinib to standard chemotherapy in 300 low-grade serous ovarian cancer (LGSOC) patients [34]. With LGSOC representing about 10% of all ovarian cancer diagnoses, chemotherapy response rates remain much lower in this group than in their high-grade counterparts. Even more so, less than 4% of recurrent LGSOC patients respond to additional chemotherapy [33]. The results of the MILO study are highly anticipated, as women with pretreated, recurrent LGSOC do not currently have a successful treatment option [35].

### Topoisomerase I inhibitors

#### Etirinotecan pegol (NKTR-102)

Etirinotecan pegol is a next-generation topoisomerase I inhibitors. Topoisomerase I inhibitors are typically semi-synthetic derivatives of the plant extract camptothecin that prevent DNA from unwinding and therefore impede tumor cells from replicating [36]. When normal topoisomerase I inhibitors like irinotecan and belotecan are quickly dispersed within the body, they not only damage healthy tissues, but also have poor half-lives, and do not sufficiently expose the tumor to the concentrated therapeutic agent. Etirinotecan pegol, instead connects small cytotoxic agents to a macromolecular polymer, using specialized linkers. These linkers are then slowly metabolized, resulting in a continuous, controlled release of the chemotherapy, which works as previously described by inhibiting topoisomerase I, and thus, hindering the division of the tumor cells. Preclinical studies have shown a 300-fold increase in the chemotherapy concentration, within the tumor, when compared to other topoisomerase I inhibitors. Along with this, increased effectiveness in tumor concentrations, the half-life of this agent has improved to 50 days, with activity in circulation throughout the entire cell cycle [37].

Clinical trials are under investigation for the use of this agent in various cancers, including ovarian cancer. One phase II, open-label study evaluated etirinotecan pegol (145 mg/m^2^), every 14 or 21 days in 71 women with platinum-resistant or refractory ovarian cancer. Of patients receiving the drug every 14 days, 27% had a RECIST response compared to 22% for those receiving it every 21 days. CA-125 responses were 61% and 52%, respectively, with a median time of 31 days to 50% decline in CA-125. The most common grade 3/4 toxicities included diarrhea (22% vs. 11%, respectively), dehydration (14% vs. 6%, respectively), and hypokalemia (14% vs. 6%, respectively) [38]. Further investigation was encouraged by the activity observed in these heavily pretreated patients.

A different randomized, multicenter, phase II trial evaluated etirinotecan pegol (145 mg/m^2^) every 14 or 21 days in 71 women with platinum-resistant or refractory ovarian cancer. Patients who received the agent every 14 days had a slightly higher response rate and response duration (20% and 4.1 months, respectively) than those who received it every 21 days (19% and 4.0 months, respectively). Median PFS and overall response rates were higher in patients receiving the drug every 21 days (5.3 and 11.7 months, respectively) than those receiving it every 14 days (4.1 and 10.0 months, respectively). The drug was well tolerated with grade 3/4 toxicities including dehydration (24%) and diarrhea (23%). Planning for a phase III investigation of etirinotecan pegol (145 mg/m^2^) every 21 days is currently underway [39]. Phase III investigation in ovarian cancer has also been encouraged by the recent, positive interim efficacy analysis in the phase III BEACON trial for metastatic breast cancer [40].

## Cytotoxic agents

### Mitotic Inhibitor

#### Paclitaxel poliglumex (CT2103)

Paclitaxel poliglumex is an agent that utilizes polyglutamate drug delivery technology, similar to that described in etirinotecan pegol above. These polyglutamate molecules are much larger than standard paclitaxel molecules, allowing them to lodge themselves in tumor tissue through leaky tumor vasculature. The drug remains inactive in the bloodstream and is too large to fit through normal vasculature, so it specifically targets only tumor cells. Once inside the tumor tissue, the agent is slowly metabolized by the tumor cells, resulting in the controlled release of the cytotoxic agent. This process reduces toxicity to healthy tissues while simultaneously increasing efficacy [41]. Paclitaxel poliglumex falls into the mitotic inhibitor drug class and is under investigation in the treatment of ovarian cancer [42].

Paclitaxel poliglumex (OPAXIO™) has completed enrollment in a 1,100 patient, randomized phase III trial comparing 12 cycles of maintenance therapy paclitaxel poliglumex or paclitaxel versus no treatment [43]. The previous phase II data that encouraged this phase III trial came nearly 10 years ago in the study of carboplatin with paclitaxel poliglumex in 82 ovarian cancer patients. The study reported 98% of patients having a major tumor response with reduction in CA-125 levels, complete responses occurring in 85% of patients, and partial response in 12%. The most common adverse events were grade 3/4 neutropenia (92%), thrombocytopenia (55%), and neuropathy (23%) [44].

### DNA minor groove binders

#### Lurbinectedin (PM1183)

Lurbinectedin is a synthetic analogue of trabectedin (previously discussed) [1]. Positive phase III results were reported for the combination of trabectedin with pegylated liposomal doxorubicin, increasing PFS and overall response rate in women with relapsed ovarian cancer [45]. Trabectedin became the first marine-derived cancer drug, derived from the colonial tunicate, *Ecteinascidia turbinate*, and marketed in Europe and Japan as Yondelis® [46]. Lurbinectedin has the same structure as trabectedin, differing only in the C subunit. Soares et al. found that the modified C subunit did not significantly alter lurbinectedin activity or cytotoxicity, and suggested the new analogue may be useful for altering dosages to increase antitumor activity [47]. Lurbinectedin works by covalently binding the minor groove in DNA. This binding causes the DNA strand to bend, increasing the incidence of double-strand breaks while also interfering with cell cycle processes and the nucleotide excision repair pathway [48].

Early *in vivo* mouse models demonstrated that single-agent lurbinectedin was effective in treating cisplatin-sensitive and cisplatin-resistant ovarian tumor models. Preclinical data also suggested that the combination of lurbinectedin with cisplatin-combined therapy was especially effective in the cisplatin-resistant tumors [49]. A randomized, phase II study of 81 platinum-resistant/refractory ovarian cancer patients compared lurbinectedin to topotecan and found that lurbinectedin had significantly improved OS (10.6 vs 5.7 months), PFS (3.9 vs 2.0 months), and the overall response rate (22%). In the lurbinectedin treatment arm, 85% of patients experienced grade 3/4 neutropenia, which was found to be preventable by using a G-CSF blood stimulating factor [50]. With this encouraging data and the success of trabectedin, lurbinectedin has received Orphan Drug status from the FDA and phase III trials in platinum-resistant patients have been planned [48, 50].

## Therapeutic vaccines

### EpCAM, CD3, and Fc receptor antibody

#### Catumaxomab

Catumaxomab (Removab®) is classified as a tri-functional antibody, with a structure comprised of an anti-EpCAM antibody and an anti-CD3 antibody. This allows catumaxomab to bind to the antigen EpCAM on tumor cells, the CD3 molecules on T cells, and to the Fc receptor on accessory cells, and in doing so, trigger an antitumor immune response [51]. Catumaxomab was approved in Europe in 2009 for the intraperitoneal treatment of malignant ascites in EpCAM-positive cancer patients, and it is currently in clinical trials in the U.S. [52]. Approximately 10% of ascites, which is the accumulation of fluid in the peritoneal cavity, is caused by cancer and called malignant ascites [53].

An open-label, phase II study of catumaxomab in patients with malignant ascites enrolled 32 women and found almost one-fourth (22.6%) of patients had at least a 400% increase in their platinum-free interval after catumaxomab treatment. Patients received catumaxomab (10, 20, 50, 150 μg) on days 0, 3, 7, and 10. The median OS was 3.6 months, with toxicities that were tolerable and consistent with what would be expected for this type of antibody [54]. Another single-arm phase II study administered one intraoperative (10 μg) and four postoperative (10, 20, 50, 150 μg) doses of catumaxomab on days 7, 10, 13, and 16. The study found a 3-year survival benefit in patients who received catumaxomab when compared to a match-pair control group (respective survival rates of 85.4% and 63.4%) [55]. This favorable survival data initiated a phase III trial of 258 EpCAM-positive cancer patients with malignant ascites [56].

## Conclusions

As the inclusion of unconventional agents are increasingly incorporated into clinical trials and practice, the hope is that drug discovery will be encouraged in all areas of cancer therapy, from improving our ability to predict response to chemotherapy, to enhancing the delivery of drugs to targeted tissues. As stated previously in an earlier review [1], with the future of cancer treatment moving towards a more personalized approach, the goal is that an individual profile will be determined, and thus agents used that target key pathways in this individual’s cancer.

## Authors’ original submitted files for images

Below are the links to the authors’ original submitted files for images. Authors’ original file for figure 1 Authors’ original file for figure 2
