# Supplementary figures and images for: Updates on drug discovery in ovarian cancer
Source: Gynecol Oncol Res Pract. 2014 Sep 30;1:3. doi: 10.1186/2053-6844-1-3 (PMC4878024; doi:10.1186/2053-6844-1-3)

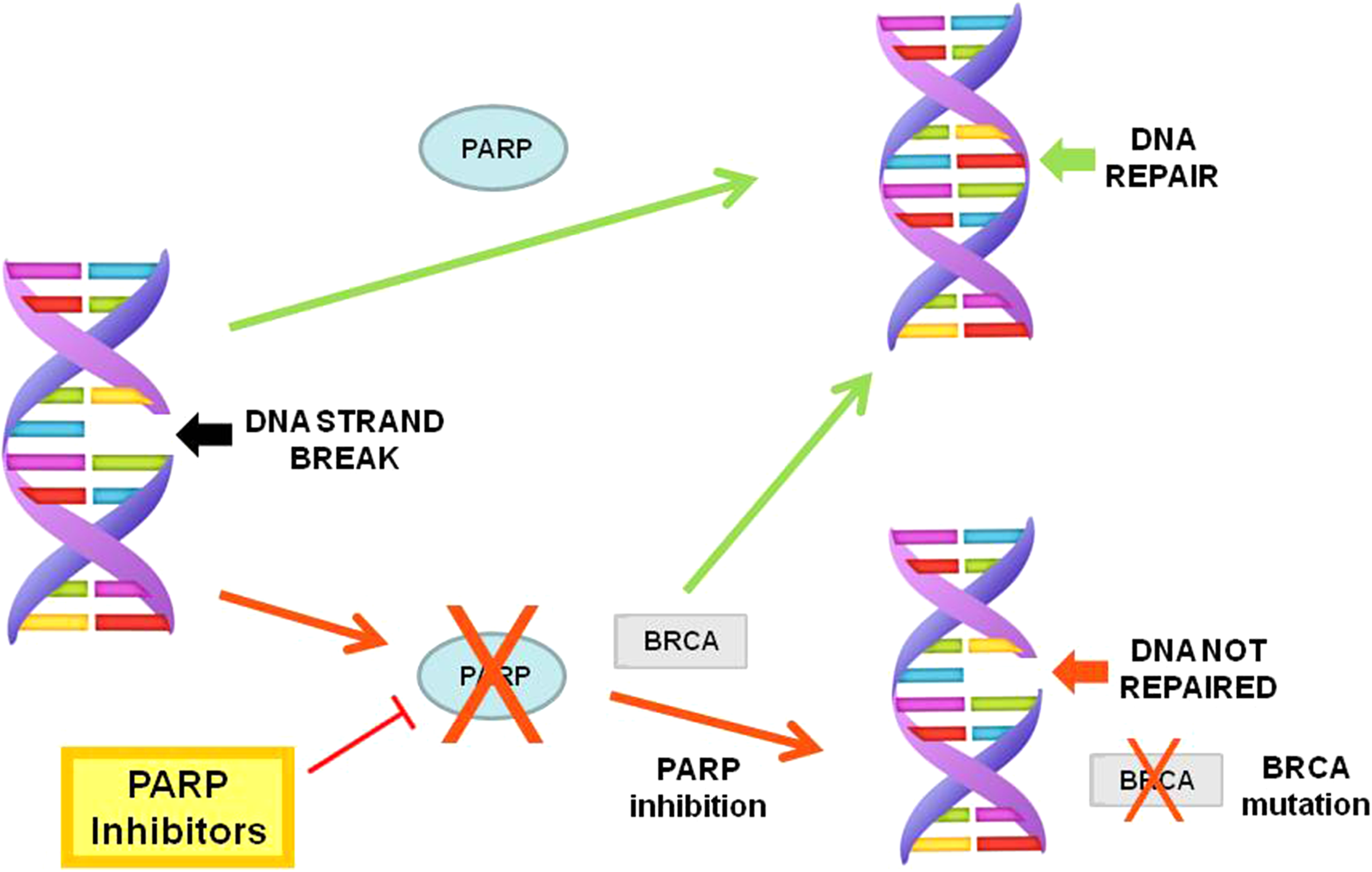

Supplement: Supplementary file 1 — Authors’ original file for figure 1 [file 40661_2014_6_MOESM1_ESM.tiff]

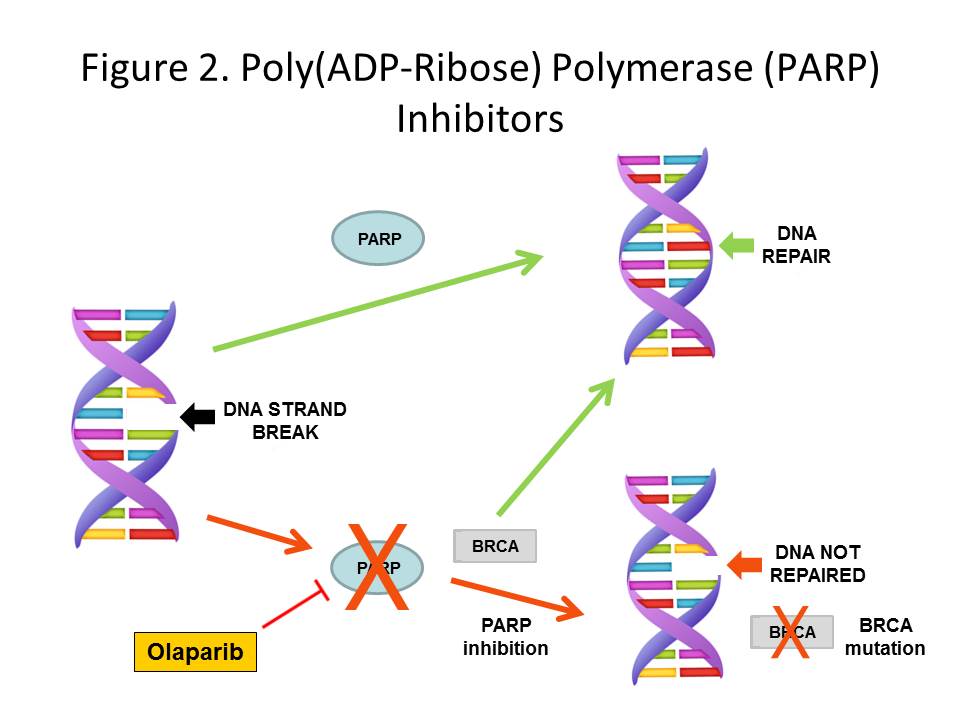

Supplement: Supplementary file 2 — Authors’ original file for figure 2 [file 40661_2014_6_MOESM2_ESM.jpeg]
